# *Passaggio* on chorists’ voice range profile: preliminary study of frequency and intensity

**DOI:** 10.1590/2317-1782/20232020266en

**Published:** 2023-09-15

**Authors:** Nathália Suellen Valeriano Cardoso, Tatiany Cíntia da Silva Brito, Adriana de Oliveira Camargo Gomes

**Affiliations:** 1 Programa de Pós-graduação em Saúde da Comunicação Humana, Universidade Federal de Pernambuco - UFPE - Recife (PE), Brasil.; 2 Departamento de Fonoaudiologia, Universidade Federal de Pernambuco - UFPE - Recife (PE), Brasil.

**Keywords:** Voice, Singing, Speech, Language and Hearing Sciences, Voice Quality, Music

## Abstract

**Purpose:**

To analyze the *passaggio* in the voice range profile of choristers, by identifying the fundamental frequencies and intensities, both in the change to the high and low registers, comparing them, by voice types.

**Method:**

67 choristers participated, mean age of 27.79 (± 7.50) years old, of the following voice types: soprano (n = 20), alto (n = 17), tenor (n = 15) and bass (n = 15). For data collection and analysis, the Vocalgram software (CTS Informática) was used, which recorded the emission of the vowel / Ɛ / in ascending and descending glissando, up to the lowest and highest note in the weakest and strongest intensities possible.

**Results:**

The values of frequencies and respective intensities of the *passaggio* were identified in ascending and descending, strong and weak emissions in all voice types. There was a higher occurrence of voice break in the high voices, compared to the low ones. The average values ​​of the frequencies found corresponded to different tones from those established in the literature for all voice types.

**Conclusion:**

The *passaggio* identified in the vocal range profile of choristers, based on their frequencies and intensities were more frequent in soprano na tenor, compared to alto and bass, in changes to the low and high registers.

## INTRODUCTION

One of the main characteristics of changes in untrained singers’ vocal register is the “voice break”, identified as *passaggio*. Despite the controversies regarding its voice type attributes, it is used as one of the parameters to classify singers’ voices^(1,2)^.

Register classification is based on the action of the predominant muscle group in the emission and its resonance effects, whether speaking or singing^(3)^. *Passaggio*, in their turn, are sudden variations in the vibratory mass in actions triggered by changes in vocal fold strain - which can be gradual, as when producing a glissando.

Transitions in the register can be identified with instrumental measures, even if the singer is skillful enough to attenuate them, leaving no audibly perceivable changes^(3,4)^.

Hence, computed acoustic measures can minimize divergences in the identification of *passaggio* notes and be a possible resource to analyze vocal behavior during register changes. This would be useful to follow up on therapy and vocal improvement results, providing visual feedback during exercises proposed to minimize such breaks - which bother singers considerably but can be disguised with constant training. Moreover, given their importance to voice classification, these notes can be better assessed with such measures, which, along with auditory-perceptual analyses, can furnish more objective data on study parameters^(5)^.

Thus, speech-language-hearing therapists are increasingly adhering to these new technologies - such as phonetography, which can assess people’s voice range profiles (VRP) through the relationship between frequency and intensity, helping analyze vocal performance^(5-7)^. In this regard, the Vocalgrama^®^ software, by CTS Informática, assesses the intensity and frequency of singing voices based on VRP records. To this end, singers emit the vowel / Ɛ / in ascending and descending glissando, from the minimum to the maximum possible tone, at strong and weak intensities^(2)^.

Besides furnishing information on voice ranges, VRP analysis generates charts that make it possible to investigate other parameters and visualize vocal changes not perceived by hearing^(8)^. These results reinforce the importance of using this instrument to record results and provide biofeedback to subjects submitted to therapy or vocal improvement.

Since the acoustic effect of transitions in registers can be disguised with constant training^(9)^, this program stands out as a great ally in speech-language-hearing practice with singers, as - among other aspects - it is useful to visually and auditorily monitor the patient’s progress. Hence, this study aimed to analyze *passaggio* notes in choir singers’ VRP, stratified by voice types, identifying their fundamental frequency (f0) and intensity, in changes to both high (ascending emissions) and low pitches (descending emissions).

## METHODS

This study was approved by the Research Ethics Committee from the Center for Health Sciences at the Federal University of Pernambuco under evaluation report no. 1.455.166. Participants signed an informed consent form before collecting data.

The sample comprised 67 adult choir singers from different choirs, divided into voice types, according to the classification given by their respective choir directors/singing teachers, namely: soprano (n = 20), contralto (n = 17), tenor (n = 15), and bass (n = 15). Their mean age was 27.79 (±7.50) years. None of the singers in the sample had voice complaints.

Data were collected and analyzed with Vocalgrama, by CTS Informática, installed in an HP Notebook PC, with Karsect HT-2 headset earphones, and an Andrea PureAudio™ USB-AS external sound card to filter and reduce noise. The voices were recorded in the program, and the microphone was adjusted 4 centimeters away from the singer’s corner of the mouth.

To record VRPs, singers were asked to emit a vowel /Ɛ/ in ascending and descending glissando, reaching the lowest and highest frequency they could produce, at weak and strong intensities. These data were collected three times from each singer to ensure measure reproducibility. The charts selected for analysis were the ones with the subjects’ best emissions.

VRP results are calculated by the program, based on a chart outlined along with the emissions. It shows the frequencies in Hertz (Hz) and intensities in decibels (dB), respectively located in the abscissa and ordinate axes^(2)^.

This study considered the following analysis variables: 1) Dependent variables: *passaggio* note identification (in %); f0 in Hertz (Hz); vocal intensity (in decibels) (dB); 2) Independent variables: /Ɛ/ glissando emission mode (strong and weak; ascending [for high-pitched records] and descending (for low-pitched records], and voice types (soprano, contralto, tenor, and bass).

**Figure 1:**
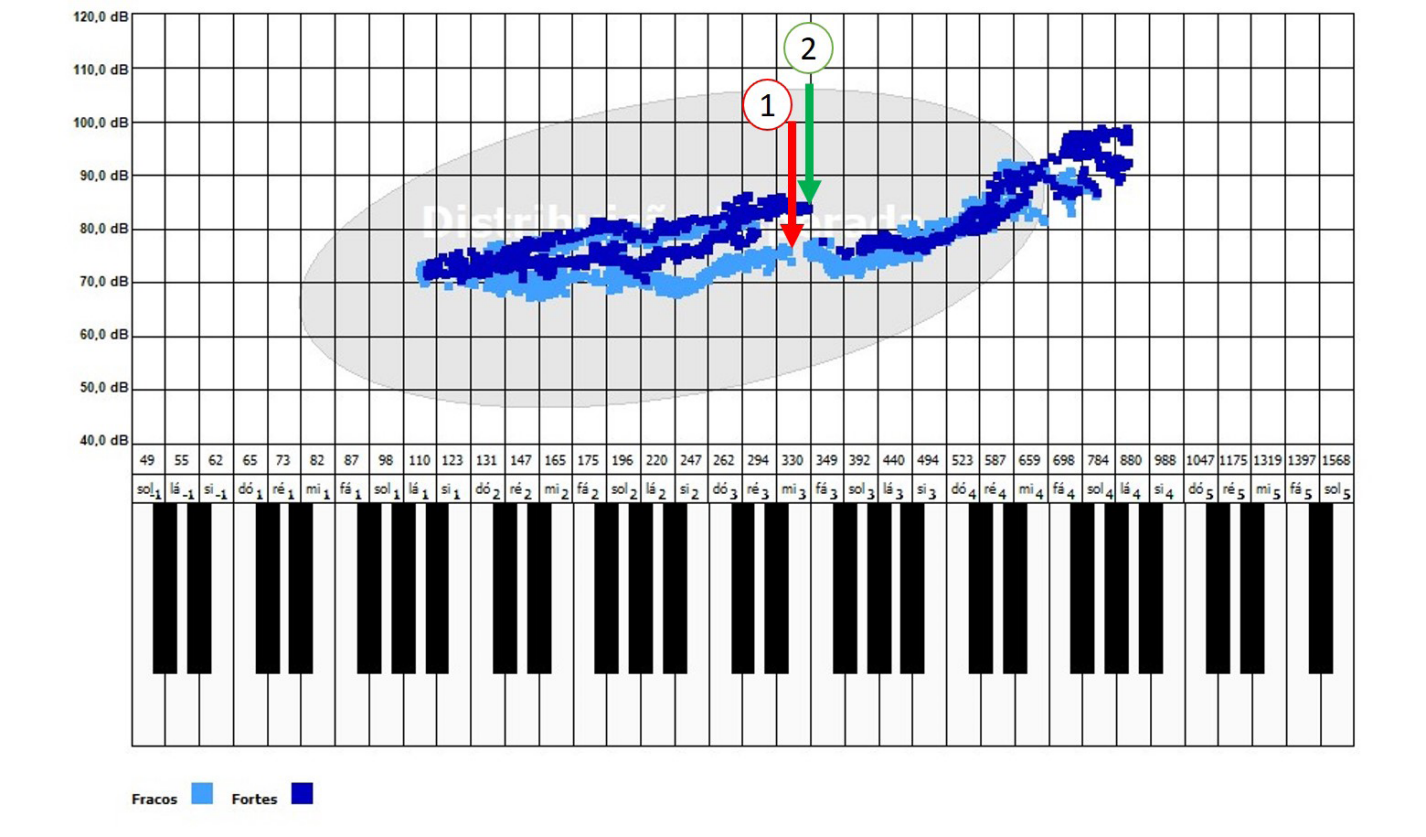
Representation of the identification of *passaggio* notes based on the discontinuity (“break”) in the chart in weak (light blue) and strong emissions (dark blue). 1 = point identified in the weak ascending emission; 2 = point identified in the strong ascending emission

The *passaggio* notes were investigated through visual and auditory-perceptual analysis of the chart generated by the software. Two researchers (speech-language-hearing therapists experienced in singing) verified the data in consensus; if they had any divergence, a third one was invited. In emission analysis, the researchers observed the charts while playing the recording of the vowel /Ɛ/ in ascending and descending glissando. The “break” was visually identified when the signal in the chart was discontinued, and auditory-perceptually identified by the typical vocal instability perceived by the researchers while they listened to the recording (Figure 1).

The presence of *passaggio* notes could be confirmed with the visual analysis of the chart generated by the program, as it represents such notes as discontinued tracing. Hence, to identify the frequency and intensity of the *passaggio* notes, the computer cursor was placed at the center of the point that corresponded to the last record before the break. In ascending emissions, this moment takes place from left to right, while in descending ones they occur from right to left (Figure 1).

The notes corresponding to the frequencies were identified with the “frequency to musical note converter”^(10)^. The musical notation used was the American one, in which the central 261.63-Hz Do in a 7-octave piano keyboard corresponds to C4.

The Shapiro-Wilk test was used to test the normality of the f0 and intensity data, rejecting the hypothesis of a normal distribution when p < 0.05. To compare the voice types regarding the percentage of *passaggio* notes identified, the chi-square test was used for the analysis of proportions between more than two independent samples, and the Mann-Whitney test was used to compare *passaggio* notes’ median f0 and intensity. All analyses were performed considering the level of significance set at 5%.

## RESULTS

The results of the percentage of subjects whose *passaggio* notes were identified through auditory-perceptual and visual analysis are shown in Table 1.

**Table 1:** Percentage distribution of subjects according to the identification of *passaggio* notes, stratified per voice type, in ascending and descending strong and weak emissions

**Voice type**	**Ascending**												**Descending**												**Asc** **X** **Des**	**Strong X weak**
	**Weak**						**Strong**						**Weak**						**Strong**							
	**I**		**NI**		**T**		**I**		**NI**		**T**		**I**		**NI**		**T**		**I**		**NI**		**T**		***p*** *	p*
	(%)	n	(%)	n	(%)	n	(%)	n	(%)	n	(%)	n	(%)	n	(%)	n	(%)	n	(%)	n	(%)	n	(%)	n		
Sp (n=20)	4.5	3	25.3	17	29.9	20	7.5	5	22.4	15	29.9	20	6.0	4	23.9	16	29.8	20	4.5	3	25.3	17	29.9	20	*0.617*	*0.315*
Co (n=17)	4.5	3	20.9	14	25.4	17	4.5	3	20.9	14	25.4	17	1.5	1	23.9	16	25.4	17	1.5	1	23.9	16	25.4	17		
Te (n=15)	13.4	9	9.0	6	22.4	15	13.4	9	9.0	6	22.4	15	6.0	4	16.4	11	22.4	15	9.0	6	13.4	9	22.4	15		
Bs (n=15)	1.5	1	20.9	14	22.4	15	4.5	3	17.9	12	22.4	15	3.0	2	19.3	13	22.4	15	7.5	5	14.9	10	22.4	15		
Total (n=67)	23.9	16	76.1	51	100.0	67	29.9	20	70.1	47	100.0	67	16.5	11	83.5	56	100.0	67	22.4	15	77.5	52	100.0	67		
p*	***0.002***						***0.034***						*0.421*						*0.072*							

* Chi-square proportion test - level of significance at 5% (p < 0.05)

**Caption:** Sp = soprano; Co = contralto; Te = tenor; Bs = bass; I = number of subjects in whom the *passaggio* (emission break) was identified; NI = number of subjects in whom the *passaggio* (emission break) was not identified; T = total; n = number of subjects; Asc X Des = comparison between occurrences of breaks in the ascending and descending emissions; Strong X Weak = comparison between occurrences of breaks in the strong and weak emissions.

In ascending emissions, there were differences in *passaggio* note identification between voice types. Breaks were more evident among tenors than in the other types in both weak and strong emissions.

In descending emissions, no differences in *passaggio* note occurrences were found between the voice types. Likewise, no differences in the percentage of *passaggio* notes were found between ascending and descending and between strong and weak emissions.

Considering the total of *passaggio* notes identified, high voice types (soprano and tenor) had greater percentages than low ones (contralto and bass). Tenors and sopranos together add up to 70% of the subjects identified.

Table 2 shows the mean frequency and intensity values found when the *passaggio* voice broke and the corresponding notes, stratified by voice type, in the ascending, descending, strong, and weak emissions.

**Table 2:** Median values of the frequencies (in Hz), and their respective notes, and intensities (in dB) found in the vocal register change in ascending and descending emissions, according to the choir singer’s voice types

**V**	**Ascending**						**Descending**						**Asc X Desc**		**Strong X Weak**	
	**Strong**			**Weak**			**Strong**			**Weak**			**f0**	**Int**	**f0**	**Int**
	**f0 (Hz)**	**Nt**	**Int** **(dB)**	**f0 (Hz)**	**Nt**	**Int** **(dB)**	**f0** **(Hz)**	**Nt**	**Int** **(dB)**	**f0 (Hz)**	**Nt**	**Int** **(dB)**	***p-value*** **a**	***p-value^a^***	***p-value^a^***	***p-value^a^***
Sp	554	C#5*	91.47	415	G#4*	72.80	587	D5*	91.20	494	B4*	78.5	*0.761*	*0.293*	*0.053*	***0.000***
n	5			3			3			4						
Co	370	F#4*	86.67	331	E4*	71.20	554	C#5*	101.1	392	G4	84				
n	3			3			1			1						
Te	311	D#4*	82.93	247	B3*	78.53	294	D4*	79.07	214	A3*	82.53				
n	9				10	9	6				9	5				
Bs	294	D4*	86.93	262	C4*	59.2	277	C#4*	93.07	211	G#3*	69.2				
n	3			1			5			2						

a Mann-Whitney test - level of significance at 5% (p < 0.05);

* Approximate correspondence

**Caption:** V = voice types; n = number of subjects; Sp = soprano; Co = contralto; Te = tenor; Bs = bass; Int = intensity; f0 = fundamental frequency; Nt = corresponding note; C = Do; D = Re; E = Mi; F = Fa; G = Sol; A = La; B = Si; # = sharp; Strong = emission as strong as possible; Asc X Desc = comparison between occurrences of breaks in the ascending and descending emissions; Strong X Weak = comparison between occurrences of breaks in strong and weak emissions.

Individual results per voice type show that *passaggio* notes are different in the ascending and descending and in the strong and weak emissions - the tenors’ and basses’ notes were quite near in the ascending emissions, and the sopranos’ and contraltos’ notes were the same in the descending emissions. However, in the inferential analysis, considering the whole group together due to the few subjects in the sample, no difference in f0 was found between ascending and descending or between strong and weak emissions. Concerning intensity, as expected, there was a difference between strong and weak emissions, but not between ascending and descending ones.

## DISCUSSION

Vocal registers are not as studied in the literature as other vocal parameters, and professionals have conceptual differences on the topic^(3)^. Computer resources can help speech-language-hearing therapists and singing teachers control transitions from one to another register, considering the visual feedback these resources provide. Thus, this study aimed to study preliminarily the characterization of *passaggio* notes by identifying their frequency and intensity in both ascending and descending transitions.

This study in 67 choir singers verified that in all emission modes, *passaggio* notes were identified in no more than 30% of the total subjects. This can be justified by the fact that the sample comprised trained singers who participated in choirs. Even though the singing practice time was not controlled, it can be assumed that more trained singers were the ones whose *passaggio* notes were not evident. To demonstrate this hypothesis, future studies should control the singing practice time.

Since emission power, projection, and control improve with better respiratory support conditions^(7,11)^, more breaks were expected to be identified in the weak emissions. On the other hand, singing practices also aim to control such support; hence, it can be inferred that singers in the sample did not have differences between these two modes thanks to their training.

It could also be explained by the sound emission form (in glissandos), which, though nearer the singing voices, may induce a greater voice range limitation than in note-to-note emissions^(3)^. A suggestion for future studies is to compare the two types of emission (in glissandos and note-to-note) to test these results.

A greater percentage of *passaggio* notes was identified in high voice types (tenors and sopranos), to which a hypothesis may be a wider phonatory range in high voices^(12)^, which would increase the odds of detecting *passaggio* notes. This hypothesis could be confirmed by comparing phonatory ranges between voice types. Hence, this study may be continued to analyze this variable.

Since few studies address *passaggio* notes’ f0, they were compared with the notes each frequency represents, also considering their applicability in voice classification. Sopranos presented notes that do not corroborate those described in the literature, namely: from Mi3 to Fa3^(13)^ (equivalent, in the American notation, to E4 and F4, respectively), or between Mi4 and Fa4^(14)^ and Fa#4^(1)^ (which, in the American notation, correspond respectively to E5, F5, and F#5).

The same happened with the contraltos, whose break is defined from Do3 to Reb3^(13)^ (corresponding to C4 to Db4, in the American notation), Re3, and from Re4 to Mi4^(14)^ (corresponding to D4, D5, and E5, respectively), and Re4^(1)^ (D5, in the American notation), while in the present study *passaggio* notes were higher in weak ascending emissions, and lower in strong descending emissions than in the cited studies.

The authors’ considered that the tenors’ *passaggio* notes ranged from Mi3 to Fa3 (E4 to F4)^(13)^ and Si#3 (B#4)^(14)^. Even though this information diverges from other authors, La2 (A3) cited as this group’s *passaggio* note^(1)^ was corroborated in the present study, which also found this note in the weak low emission. Contrarily, Re3 (D4) was present in the weak ascending emission.

The basses’ notes in the weak and strong ascending emissions were respectively Do3 (C4) and Re3 (D4), while the ones in the weak and strong descending emissions were La2 (A3) and Do3 (C4). This finding differs from Mib3^(1)^ (Eb4) but agrees partly with the study that admits that the “break” can be found from Do3 to Reb3 (C4 to Db4)^(13)^. By considering Mi#2 (E#3), Sol2 (G3), Re3 (D4), and Sol3 (G4) as this voice type’s *passaggio* notes^(14)^, the findings in this study corroborate in part such propositions.

Despite the differences in note analysis, the assessment per octave showed that notes identified from the four voice types belong to the indicated octaves: the third and fourth octaves in sopranos and contraltos; the third octave and the transition between the third and fourth octaves in tenors; and second and third octaves in basses^(14)^. Only La2 (A3), found in the tenors’ weak descending emissions, belongs to the second octave and, therefore, was not expected.

Concerning divergences between this study and the literature, the analysis method may have interfered, as the collection used a voice range test, in which choir singers emit from low to high notes and vice-versa. Thus, future studies should use the central Do as the starting point for ascending and descending emissions.

No comparative data was found in the literature regarding the intensity of notes in the weak and strong emissions, and the number of subjects per voice type whose *passaggio* notes were identified was not enough for statistical comparisons. Therefore, future studies should have larger samples to compare differences in this variable between voice types.

*Passaggio* notes were identified in all voice types in both weak and strong emissions. Median frequency values corresponded to notes different from those established in the literature for all voice types. As for *passaggio* note intensity, studies with larger samples are needed to establish reference values.

This preliminary study also demonstrated Vocalgrama’s applicability to identify instrumentally the physiological phenomenon of *passaggio* notes. The visual chart feedback of this event may help both classify voices and follow up on treatment or vocal improvement results in singers and non-singers. Hence, it is useful as a voice analysis record and as a therapy resource.

Moreover, this study differs from other ones in important aspects, namely: 1) it took frequency measures, rather than using musical instruments, making data more precise and allowing for the calculation of means and medians; 2) with glissando instead of note-to-note emissions because glissando emissions are comparatively nearer the singing voice; 3) in the strong and weak emissions, which in the singing voice have an essential value in aerodynamic control and musical expressiveness.

It must be also highlighted that differences on the topic are evident in the literature^(12-15)^, demonstrating that there is no consensus between authors. The lack of studies whose analyses consider different emission conditions - such as ascending, descending, strong, and weak - also hinders analyses per note or frequency.

Hence, given the current possibility of new and less subjective analysis instruments than those traditionally used, this study should be continued with a larger sample, comparing trained and untrained singers, and comparing instrumental data with auditory-perceptual analysis results.

## CONCLUSION

*Passaggio* notes identified in choir singers’ VRP based on their frequencies and intensities occurred more often in sopranos and tenors than in contraltos and basses in the changes to low and high registers.

## References

[1] Paparotti C, Leal V (2011). Cantonário: guia prático para o canto..

[2] Lima AT, Lucena JA, Araújo ANB, Lira ZS, Gomes AOC (2016). Perfil de extensão vocal em coristas após técnica de vibração de língua associada a escalas. Rev CEFAC.

[3] Roubeau B, Henrich N, Castellengo M (2009). Laryngeal vibratory mechanisms: the notion of vocal register revisited. J Voice.

[4] Henrich N (2006). Mirroring the voice from Garcia to the present day: some insights into singing voice registers. Logoped Phoniatr Vocol.

[5] Camargo TF, Barbosa DA, Teles LCS (2007). Características da fonetografia em coristas de diferentes classificações vocais. Rev Soc Bras Fonoaudiol.

[6] Vargas AC, Costa AG, Hanayama EM (2005). Perfil de extensão vocal em indivíduos falantes normais do português brasileiro. Rev CEFAC.

[7] Šiupšinskienė N, Lycke H (2011). Effects of vocal training on singing and speaking voice characteristics in vocally healthy adults and children based on choral and nonchoral data. J Voice.

[8] Santos ACM, Borrego MCM, Behlau M (2015). Efeito de treinamento vocal direto e indireto em estudantes de Fonoaudiologia. CoDAS.

[9] Behlau M, Madázio G (2015). Voz: tudo o que você queria saber sobre fala e canto.

[10] Botros A http://newt.phys.unsw.edu.au/music/note/.

[11] Gava  WG, Ferreira LP, Silva MAA (2010). Apoio respiratório na voz cantada: perspectiva de professores de canto e fonoaudiólogos. Rev CEFAC.

[12] Storck C, Unteregger F (2018). Cricothyroid joint type as predictor for vocal fold elongation in professional singers. Laryngoscope.

[13] Martinez E (2000). Regência coral: princípios básicos.

[14] Costa PJBM, Ferreira KL, Camargo ZA, Pinho SM (2006). Extensão vocal de cantores de coros evangélicos amadores. Rev CEFAC.

[15] Herbst CT, Duus E, Jers H, Švec JG (2012). Quantitative voice class assessment of amateur choir singers: a pilot investigation. Int J Res Chor Sing..

