# Supplementary material for: Passaggio on chorists’ voice range profile: preliminary study of frequency and intensity
Source: Codas. 2023 Sep 15;35(4):e20200266. doi: 10.1590/2317-1782/20232020266en (PMC10546917; doi:10.1590/2317-1782/20232020266en)
Supplement: Translation

# Translation: Nota de passagem no perfil de extensão vocal de coristas: estudo preliminar de frequência e intensidade

**DOI:** 10.1590/2317-1782/20232020266pt

**Authors:** Nathália Suellen Valeriano Cardoso, Tatiany Cíntia da Silva Brito, Adriana de Oliveira Camargo Gomes

**Affiliations:** 1 Programa de Pós-graduação em Saúde da Comunicação Humana, Universidade Federal de Pernambuco - UFPE - Recife (PE), Brasil.; 2 Departamento de Fonoaudiologia, Universidade Federal de Pernambuco - UFPE - Recife (PE), Brasil.

**Keywords:** Voz, Canto, Fonoaudiologia, Qualidade da Voz, Música

## Abstract

**Objetivo:**

Analisar a nota de passagem no perfil de extensão vocal de coristas, pela identificação das frequências fundamentais e intensidades, tanto na mudança para o registro agudo quanto para o grave, comparando-as, por naipe.

**Método:**

Participaram 67 coristas, com média de idade de 27,79 (±7,50) anos, dos naipes: soprano (n=20), contralto (n=17), tenor (n=15) e baixo (n=15). Para coleta e análise dos dados utilizou-se o software *Vocalgrama* (CTS Informática) no qual foi realizada a gravação da emissão da vogal /Ɛ/ em *glissando* ascendente e descendente, até às frequências mais grave e mais aguda, nas intensidades mais fraca e mais forte possíveis.

**Resultados:**

Foram identificados os valores das frequências e respectivas intensidades das notas de passagem nas emissões ascendentes e descendentes fortes e fracas nos quatro naipes. Houve maior ocorrência de identificação de quebra nas vozes agudas, comparativamente às mais graves. Os valores médios das frequências encontradas foram correspondentes a notas diferentes das estabelecidas na literatura para todos os naipes.

**Conclusão:**

As notas de passagem identificadas no perfil de extensão vocal de coristas, a partir de suas frequências e intensidades, obtiveram maior ocorrência nos naipes soprano e tenor, comparativamente ao contralto e baixo, nas mudanças para os registros graves e agudos.

## INTRODUÇÃO

Uma das principais características da mudança de registro do cantor não treinado é a “quebra na voz”, identificada como nota de passagem. A despeito das controvérsias quanto às suas atribuições aos naipes, ela é utilizada como um dos parâmetros para a classificação vocal de cantores^(1,2)^.

A classificação dos registros tem como base a ação do grupo muscular predominante na emissão, bem como seus efeitos de ressonância, seja na voz falada ou cantada^(3)^. As notas de passagem, por sua vez, representam variações repentinas da massa vibratória em ações ocasionadas por modificações na tensão das pregas vocais, que podem ser de forma gradual, como durante a produção de um glissando.

A transição no registro pode ser identificada por medidas instrumentais, ainda que o cantor seja habilidoso o suficiente para suavizá-la, sem que sejam produzidas mudanças auditivamente perceptíveis^(3,4)^.

Desse modo, o uso de medidas acústicas computadorizadas poderia minimizar as divergências quanto à identificação das notas de passagem, além de ser um possível recurso para a análise do comportamento vocal durante as mudanças de registro, para acompanhamento de resultados terapêuticos e do aprimoramento vocal, permitindo o *feedback* visual durante a execução dos exercícios propostos para minimização dessas quebras, tão incômodas para o cantor, mas que podem ser ocultadas com treino constante. Além disso, devido à sua importância para a classificação vocal, essas notas podem ser melhor avaliadas com tais medidas que, somadas às análises perceptivo-auditivas, são capazes de fornecer dados mais objetivos sobre os parâmetros estudados^(5)^.

Por isso, a adesão dos fonoaudiólogos a essas novas tecnologias é crescente, como ocorre com a fonetografia, capaz de avaliar o perfil de extensão vocal (PEV) dos indivíduos por meio da relação entre frequência e intensidade, favorecendo a análise da performance vocal^(5-7)^. Nesse sentido, o Vocalgrama® da CTS Informática é um *software* que pode ser utilizado para avaliar a intensidade e frequência da voz cantada pelo registro do perfil de extensão vocal (PEV). Para tal, emite-se a vogal / Ɛ / em glissando ascendente e descendente, da tonalidade mínima à máxima possível, nos níveis de intensidade fraco e forte^(2)^.

Além de fornecer informações sobre a extensão vocal, a análise gerada pelo PEV resulta em gráficos que propiciam a investigação de outros parâmetros, além de auxiliar na visualização de mudanças vocais não percebidas auditivamente^(8)^. Tais resultados reforçam a importância do uso desse instrumento para o registro de resultados e para o *biofeedback* dos sujeitos submetidos à terapia ou aprimoramento vocais.

Considerando-se que os efeitos acústicos da transição de registros podem ser ocultados com o treino constante^(9)^, este programa desponta como um grande aliado na atuação fonoaudiológica com cantores, pois - dentre outras coisas - é útil no monitoramento visual e auditivo da evolução do paciente. Desse modo, o objetivo deste trabalho foi analisar a nota de passagem no perfil de extensão vocal de coristas, identificando a frequência fundamental e a intensidade, tanto na mudança para o registro agudo (emissões ascendentes), quanto para o grave (emissões descendentes), em coristas, estratificados por naipes.

## MÉTODO

Este trabalho foi aprovado pelo Comitê de Ética em Pesquisa do Centro de Ciências da Saúde da Universidade Federal de Pernambuco, sob o parecer 1.455.166. Os participantes assinaram o Termo de Consentimento Livre e Esclarecido, antes da coleta dos dados.

Participaram 67 coristas adultos de diferentes corais, divididos em naipes, segundo a classificação dada por seus respectivos regentes/professores de canto, a saber: soprano (n=20), contralto (n=17), tenor (n=15) e baixo (n=15). A média de idade foi de 27,79 (±7,50) anos. Toda a amostra foi composta por cantores sem queixas vocais.

Para coleta e análise dos dados utilizou-se o *software* Vocalgrama, da CTS Informática, instalado em um computador HP Notebook PC, com microfone Auricular Karsect HT-2 e um Adaptador Andrea PureAudio™ USB-AS, para filtragem e redução dos ruídos. Os registros vocais foram gravados no próprio programa, e o microfone foi ajustado a quatro centímetros da comissura labial do cantor.

Para a gravação do PEV, o cantor foi solicitado a emitir a vogal /Ɛ/ em glissando ascendente e descendente, chegando à frequência mais grave e a mais aguda que era capaz de produzir, nas intensidades fraca e forte. Esses dados foram coletados três vezes, para cada cantor, a fim de se garantir a reprodutibilidade das medidas. O gráfico selecionado para análise foi sempre o que representava a melhor emissão do sujeito.

Os resultados do PEV são calculados pelo próprio programa, a partir de um gráfico, delineado à medida em que a emissão é realizada. Nele é possível identificar as frequências em Hertz (Hz) e as intensidades em decibel (dB), localizadas no eixo das abscissas e ordenadas, respectivamente^(2)^.

Para este estudo, foram consideradas as seguintes variáveis de análise: 1) *Variáveis dependentes:* identificação das notas de passagem (em %); frequência fundamental (f0) em Hertz (Hz); intensidade vocal em decibel (dB); 2) *Variáveis independentes:* Modo de emissão do/Ɛ/ glissando (forte e fraco; ascendente (para o registro agudo) e descendente (para o registro grave) e naipes (soprano, contralto, tenor e baixo).

A investigação da nota de passagem foi realizada através da análise do gráfico gerado pelo software, de forma visual e perceptivo-auditiva. Os dados foram averiguados por dois pesquisadores (fonoaudiólogos com experiência em canto), em consenso, sendo um terceiro convocado quando houve divergência entre os dois primeiros. Os pesquisadores observavam o gráfico, enquanto reproduziam a gravação da vogal /Ɛ/ em glissando (nos momentos ascendente e descendente) durante a análise das emissões. A “quebra” era identificada visualmente no momento em que ocorria a descontinuidade do sinal no gráfico e, de forma perceptivoauditiva, pela instabilidade vocal típica percebida pelos pesquisadores, enquanto ouviam tal reprodução (Figura 1).

**Figura 1:** Representação da identificação da nota de passagem, a partir da descontinuidade (“quebra”) no gráfico, nas emissões fraca (azul claro) e forte (azul escuro). 1= ponto identificado na emissão ascendente, fraca; 2 = ponto identificado na emissão ascendente, forte

A confirmação da presença da nota de passagem foi possível pela análise visual do gráfico gerado pelo programa, pois este as representa como uma descontinuidade no traçado produzido. Dessa forma, para identificação da frequência e intensidade das notas de passagem, posicionou-se o cursor do computador no centro do ponto que corresponde ao último registro anterior à quebra. Para as emissões ascendentes, esse momento ocorre da esquerda para direita, enquanto que para as descendentes, da direita para a esquerda (Figura 1).

Para identificação das notas correspondentes às frequências encontradas utilizou-se o “frequency to musical note converter”^(10)^. A notação musical utilizada foi segundo a notação americana, em que o Dó central (de 261,63 Hz) do teclado de um piano de sete oitavas, corresponde a C4.

Para testar a normalidade dos dados de f0 e intensidade, foi utilizado o teste de Shapiro-Wilk, rejeitando-se a hipótese de distribuição normal quando p<0,05. Para comparação entre os naipes, quanto à porcentagem de notas de passagem identificadas, utilizou-se o teste Qui-Quadrado para análise de proporções entre mais de duas amostras independentes e para a comparação entre as medianas de f0 e de intensidade das notas de passagem, utilizou-se o teste *Mann-Whitney*. Todas as análises foram realizadas considerando-se o nível de significância de 5%.

## RESULTADOS

Os resultados quanto à porcentagem de sujeitos cujas notas de passagem foram identificadas, por meio da análise perceptivo-auditiva e visual, estão expressos na Tabela 1.

**Tabela 1:** Distribuição, em porcentagem, dos sujeitos segundo a identificação da nota de passagem, estratificados por naipe, nas emissões ascendentes e descendentes e nos modos de emissão fraco e forte

**Naipe**	**Ascendentes**												**Descendentes**												**Asc X Des**	**Forte X Fraco**
	**Fraco**						**Forte**						**Fraco**						**Forte**							
	**I**		**NI**		**T**		**I**		**NI**		**T**		**I**		**NI**		**T**		**I**		**NI**		**T**		p*	p*
	**(%)**	**n**	**(%)**	**n**	**(%)**	**n**	**(%)**	**n**	**(%)**	**n**	**(%)**	**n**	**(%)**	**n**	**(%)**	**n**	**(%)**	**n**	**(%)**	**n**	**(%)**	**n**	**(%)**	**n**		
Sp (n=20)	4,5	3	25,3	17	29,9	20	7,5	5	22,4	15	29,9	20	6,0	4	23,9	16	29,8	20	4,5	3	25,3	17	29,9	20	*0,617*	*0,315*
Co (n=17)	4,5	3	20,9	14	25,4	17	4,5	3	20,9	14	25,4	17	1,5	1	23,9	16	25,4	17	1,5	1	23,9	16	25,4	17		
Te (n=15)	13,4	9	9,0	6	22,4	15	13,4	9	9,0	6	22,4	15	6,0	4	16,4	11	22,4	15	9,0	6	13,4	9	22,4	15		
Bx (n=15)	1,5	1	20,9	14	22,4	15	4,5	3	17,9	12	22,4	15	3,0	2	19,3	13	22,4	15	7,5	5	14,9	10	22,4	15		
Total (n=67)	23,9	16	76,1	51	100,0	67	29,9	20	70,1	47	100,0	67	16,5	11	83,5	56	100,0	67	22,4	15	77,5	52	100,0	67		
***p*** *	***0,002***						***0,034***						*0,421*						*0,072*							

* Teste *Qui-quadrado para análise de proporções* - nível de significância a 5% (p<0,05)

**Legenda:** Sp = soprano; Co = contralto; Te = tenor; Bx = baixo; I = quantidade de sujeitos em que foi identificado o momento de passagem (quebra na emissão); NI = quantidade de sujeitos em que não se identificou o momento de passagem (quebra na emissão); T = total; n = número de sujeitos; Asc X Des = comparação entre as ocorrências de quebra nas emissões ascendentes e nas emissões descendentes; Forte X Fraco = comparação entre as ocorrências de quebra nas emissões forte e fraca.

Quanto às emissões no modo ascendente, houve diferença entre os naipes quanto à identificação das notas de passagem e no grupo dos tenores as quebras foram mais evidentes, comparativamente aos demais naipes, tanto nas emissões fracas, quanto nas fortes.

Em relação às emissões no modo descendente, não houve diferença entre os naipes quanto à ocorrência das notas de passagem. Na comparação entre os modos ascendente e descendente e entre as emissões forte e fraca, não houve diferenças na porcentagem de identificação das notas de passagem.

Ao considerar o total das notas de passagem identificadas, a maior porcentagem está nos naipes caracterizados pelas vozes mais agudas (soprano e tenor) comparativamente aos das vozes graves (contralto e baixo). A soma de tenores e sopranos representa 70% dos sujeitos identificados.

A Tabela 2 apresenta a média dos valores da frequência e intensidade, encontradas no momento da quebra de passagem de registro vocal, e as notas correspondentes, estratificados por naipe, nas emissões ascendentes e descendentes, fraco e forte.

**Tabela 2:** Valores medianos das frequências (em Hz), e suas respectivas notas, e das intensidades (em dB) encontrados no momento da mudança de registro vocal, nas emissões ascendentes e descendentes, segundo os naipes dos coristas

**N**	**Ascendentes**						**Descendentes**						**Asc X Desc**		**Forte X Fraco**	
	**Forte**			**Fraco**			**Forte**			**Fraco**			**f0**	**Int**	**f0**	**Int**
	**f0 (Hz)**	**Nt**	**Int (dB)**	**f0 (Hz)**	**Nt**	**Int (dB)**	**f0 (Hz)**	**Nt**	**Int (dB)**	**f0 (Hz)**	**Nt**	**Int (dB)**	***p-valor*** ^a^	***p-valor^a^***	***p-valor^a^***	***p-valor^a^***
Sp	554	C#5*	91,47	415	G#4*	72,80	587	D5*	91,20	494	B4*	78,5	*0,761*	*0,293*	*0,053*	***0,000***
n	5			3			3			4						
Co	370	F#4*	86,67	331	E4*	71,20	554	C#5*	101,1	392	G4	84				
n	3			3			1			1						
Te	311	D#4*	82,93	247	B3*	78,53	294	D4*	79,07	214	A3*	82,53				
n	9				10	9	6				9	5				
Bx	294	D4*	86,93	262	C4*	59,2	277	C#4*	93,07	211	G#3*	69,2				
n	3			1			5			2						

a Teste *Mann-Whitney* - nível de significância a 5% (p<0,05);

* correspondência aproximada

**Legenda:** N = naipes; n = número de sujeitos; Sp = soprano; Co = contralto; Te = tenor; Bx = baixo; Int = intensidade; f0 = frequência fundamental; Nt = nota correspondente; C = Dó; D = Ré; E = Mi; F = Fá; G = Sol; A = Lá; B = Si; # = sustenido; Forte = modo de emissão o mais forte possível; Asc X Desc = comparação entre as ocorrências de quebra nas emissões ascendentes e nas emissões descendentes; Forte X Fraco = comparação entre as ocorrências de quebra nas emissões forte e fraca.

Ao considerar os resultados individuais, por naipe, observa-se que as notas de passagem são diferentes nos momentos ascendentes e descendentes e nos modos fraco e forte, sendo que as notas dos tenores e baixos foram muito próximas, nas emissões ascendentes e a de sopranos e contraltos foi a mesma, nas emissões descendentes. No entanto, na análise inferencial, em que o grupo foi considerado em sua totalidade, devido ao número pequeno de sujeitos na amostra, não houve diferença na f0 entre as emissões ascendentes e descendentes e entre os momentos fraco e forte. Quanto à intensidade, como esperado, houve diferença entre os momentos fraco e forte, mas não entre as emissões ascendentes e descendentes.

## DISCUSSÃO

O estudo dos registros vocais não é tão salientado na literatura, comparativamente aos demais parâmetros vocais, além de apresentar divergências conceituais entre profissionais^(3)^. Recursos computadorizados podem ajudar fonoaudiólogos e professores de canto no controle das transições entre um registro e outro, considerando-se o feedback visual que esses recursos permitem. Portanto, este estudo se propôs a estudar de forma preliminar a caracterização da nota de passagem por meio da identificação da frequência e intensidade, tanto na transição de notas ascendentes quanto descendentes.

Neste estudo, com 67 coristas, pôde-se notar que, em todos os modos de emissão, a identificação das notas de passagem não ultrapassou 30% do total de sujeitos. Isso pode se justificar pelo fato de que a amostra é constituída de cantores treinados, por serem participantes de Coral. A despeito de não se ter controlado a variável tempo de treinamento em canto, pode-se supor que os cantores mais treinados tenham sido os que não apresentaram a nota de passagem evidenciada. Para a comprovação dessa hipótese, sugere-se controlar a variável tempo de treinamento em canto, em estudos futuros.

Uma vez que a potência, projeção e controle da emissão são aprimorados quando se obtêm melhores condições de apoio respiratório^(7,11)^, esperava-se maior identificação da quebra nas emissões fracas. Por outro lado, considerando-se que o treino do canto visa também o controle desse apoio, pode-se inferir que, na amostra estudada, os cantores não apresentaram diferenças entre esses dois modos, em consequência de seu treino.

Outra possível explicação estaria na forma de emissão dos sons (em glissandos) que, a despeito de serem emissões mais próximas da voz cantada, podem induzir à maior limitação da extensão vocal, comparativamente à emissão nota a nota^(3)^. Uma sugestão para futuros estudos é a de se comparar os dois tipos de emissão: em glissando e nota a nota, para se testar esses resultados.

Quanto à distribuição entre os naipes da ocorrência de identificação das notas de passagem, cuja maior porcentagem ocorreu nos naipes de vozes agudas (tenores e sopranos) uma hipótese para tal seria a possível maior extensão fonatória das vozes agudas^(12)^, que aumentariam as chances de evidenciação da nota de passagem. Essa hipótese poderia ser confirmada por meio da comparação das extensões fonatórias entre os naipes. Sugere-se portanto, a continuidade deste estudo, para análise dessa variável.

Devido à escassez de estudos que levem em consideração a frequência fundamental das notas de passagem, optou-se por compará-las às notas que cada frequência representa, tendo em vista, também, sua aplicabilidade na classificação vocal. O naipe dos sopranos apresentou notas que não corroboram as descritas na literatura, a saber: de Mi3 a Fa3^(13)^ (equivalente, na notação americana, a E4 e F4, respectivamente), ou entre Mi4 a Fa4^(14)^ e Fa#4^(1)^ (que, na notação americana, correspondem a E5, F5 e F#5, respectivamente).

O mesmo aconteceu com o grupo dos contraltos, cuja quebra é definida de Do3 a Reb3^(13)^ (correspondente a C4 a Db4, na notação americana), em Re3 e entre Re4 e Mi4^(14)^ (correspondente a D4, D5 e E5, respectivamente) e em Ré4^(1)^ (D5, na notação americana), enquanto que no presente estudo a nota de passagem foi mais aguda nas emissões ascendentes, no modo fraco; e mais grave nas emissões descendentes, no modo forte, comparativamente aos estudos citados.

Para os tenores, os autores assumem que as notas de passagem vão de Mi3 a Fa3 (E4 a F4)^(13)^ e Si#3 (B#4)^(14)^. Embora sendo um dado que diverge dos demais autores, o La2 (A3) citado como a nota de passagem para esse grupo^(1)^ foi corroborado pelo presente estudo, que também encontrou essa nota na emissão grave fraca. O mesmo não aconteceu com a nota Ré3 (D4), presente na emissão ascendente fraca.

No naipe dos baixos, as notas das emissões ascendentes fraca e forte foram Dó3 (C4) e Ré3 (D4), respectivamente, ao passo que aquelas encontradas nos descendentes fraco e forte foram Lá2 (A3) e Dó3 (C4). Tal achado difere do Mib3^(1)^ (Eb4), mas concorda em parte com o estudo que admite que a “quebra” pode ser encontrada de Dó3 a Reb3 (C4 a Db4)^(13)^. Ao assumir Mi#2 (E#3), Sol2 (G3), Ré3 (D4) e Sol3 (G4) como notas de passagem para esse naipe^(14)^, os achados do presente estudo corrobora, em parte, tais proposições.

Ainda que apresente diferenças na análise das notas, a avaliação por oitava permitiu observar que as notas identificadas para as quatro vozes pertencem às oitavas indicadas: terceira e quarta oitava, para sopranos e contraltos, terceira oitava e a transição entre a terceira e a quarta oitavas para os tenores, e segunda e terceira oitavas para os baixos^(14)^. Apenas o Lá2 (A3), encontrado na emissão descendente fraca do tenor, encontra-se na segunda oitava e, portanto, fora do que seria esperado.

Para as divergências encontradas entre este estudo e a literatura, pode-se inferir que o método de análise tenha interferido, por serem coletados em um teste de extensão vocal, no qual o corista vai da emissão mais grave à mais aguda e vice-versa. Sugere-se, portanto, que estudos futuros adotem o Dó central como ponto inicial para as emissões ascendentes e descendentes.

Quanto à intensidade das notas, nas emissões fracas e fortes, não há dados comparativos na literatura e o número de sujeitos, por naipe, cujas notas de passagem foram identificadas, não permitiu a comparação estatística. Portanto, sugere-se aumento da amostra para estudos futuros, no intuito de se comparar a diferença dessa variável, por naipe.

Portanto, as notas de passagem foram identificadas em todos os naipes, tanto nas emissões fracas, quanto nas emissões fortes. Os valores medianos das frequências encontradas foram correspondentes a notas diferentes das estabelecidas na literatura, para todos os naipes. Quanto às intensidades das notas de passagem, há necessidade de estudos com amostras maiores para o estabelecimento de valores de referência.

Ressalte-se que este estudo preliminar permitiu ainda elucidar a aplicabilidade do Vocalgrama na identificação, de forma instrumental, do fenômeno fisiológico da nota de passagem. O *feedback* visual gráfico desse evento pode auxiliar tanto na classificação vocal quanto no acompanhamento de resultados de tratamento ou de aprimoramento vocal de cantores e não cantores, sendo útil para o registro da análise da voz e como recurso terapêutico.

Ademais, o presente estudo se diferencia dos demais em pontos importantes, a saber: 1) realizou medidas de frequência, em vez de uso de instrumentos musicais, tornando os dados mais precisos e possíveis de serem calculadas as médias e medianas; 2) com emissões em glissando, em vez de nota a nota, por ser a emissão em glissando mais próxima da produção de voz cantada, comparativamente; 3) nos modos de emissão forte e fraco, que, na voz cantada, têm um valor imprescindível no controle aerodinâmico e na expressividade musical.

Vale ressaltar, ainda, que as diferenças sobre o tema são evidentes na própria literatura^(12-15)^, demonstrando que não há um consenso entre os autores. A carência de estudos que façam uma análise considerando as diferentes condições de emissão, como ascendente e descendente, forte e fraco, também dificultam a análise por nota ou frequência.

Portanto, tendo em vista a possibilidade atual de novos instrumentos de análise de caráter menos subjetivo, como os utilizados tradicionalmente, sugere-se a continuidade do estudo com o aumento da amostra, a comparação de cantores treinados e não treinados e a comparação dos dados instrumentais com os resultados de análise perceptivoaudititva.

## CONCLUSÃO

As notas de passagem identificadas no perfil de extensão vocal de coristas, a partir de suas frequências e intensidades, obtiveram maior ocorrência nos naipes soprano e tenor, comparativamente ao contralto e baixo, nas mudanças para os registros graves e agudos.
